# Mapping gastrointestinal gene expression patterns in wild primates and humans via fecal RNA-seq

**DOI:** 10.1186/s12864-019-5813-z

**Published:** 2019-06-14

**Authors:** Ashok Kumar Sharma, Barbora Pafčo, Klára Vlčková, Barbora Červená, Jakub Kreisinger, Samuel Davison, Karen Beeri, Terence Fuh, Steven R. Leigh, Michael B. Burns, Ran Blekhman, Klára J. Petrželková, Andres Gomez

**Affiliations:** 1 Department of Animal Science, University of Minnesota, Twin Cities, USA; 2 The Czech Academy of Sciences, Institute of Vertebrate Biology, Květná 8, 603 65 Brno, Czech Republic; 3 Department of Pathology and Parasitology, Faculty of Veterinary Medicine, University of Veterinary and Pharmaceutical Sciences Brno, Palackého tř. 1946/1, 612 42 Brno, Czech Republic; 4 Department of Zoology, Faculty of Science, Charles University, Viničná 7, 128 44 Praha, Czech Republic; 5 Vanderbilt University medical center Technologies for Advanced Genomics, Vanderbilt University medical center, Nashville, TN USA; 6 WWF Central African Republic, Bangui, Central African Republic; 7 Department of Anthropology, University of Colorado, Boulder, CO USA; 8 Loyola University Chicago, Quinlan Life Sciences Building, Chicago, IL USA; 9 Department of Genetics, Cell Biology, and Development, University of Minnesota, Twin Cities, MN USA; 10 Department of Ecology, Evolution and Behavior, University of Minnesota, Twin Cities, MN USA; 11 The Czech Academy of Sciences, Biology Centre, Institute of Parasitology, Branišovská 31, 370 05 České Budějovice, Czech Republic; 12 Liberec Zoo, Lidové sady 425/1, 460 01 Liberec, Czech Republic

**Keywords:** Gene expression, Nonhuman primate, RNA-seq, Noninvasive method

## Abstract

**Background:**

Limited accessibility to intestinal epithelial tissue in wild animals and humans makes it challenging to study patterns of intestinal gene regulation, and hence to monitor physiological status and health in field conditions. To explore solutions to this limitation, we have used a noninvasive approach via fecal RNA-seq, for the quantification of gene expression markers in gastrointestinal cells of free-range primates and a forager human population. Thus, a combination of poly(A) mRNA enrichment and rRNA depletion methods was used in tandem with RNA-seq to quantify and compare gastrointestinal gene expression patterns in fecal samples of wild *Gorilla gorilla gorilla* (*n* = 9) and BaAka hunter-gatherers (*n* = 10) from The Dzanga Sangha Protected Areas, Central African Republic.

**Results:**

Although only a small fraction (< 4.9%) of intestinal mRNA signals was recovered, the data was sufficient to detect significant functional differences between gorillas and humans, at the gene and pathway levels. These intestinal gene expression differences were specifically associated with metabolic and immune functions. Additionally, non-host RNA-seq reads were used to gain preliminary insights on the subjects’ dietary habits, intestinal microbiomes, and infection prevalence, via identification of fungi, nematode, arthropod and plant RNA.

**Conclusions:**

Overall, the results suggest that fecal RNA-seq, targeting gastrointestinal epithelial cells can be used to evaluate primate intestinal physiology and gut gene regulation, in samples obtained in challenging conditions in situ. The approach used herein may be useful to obtain information on primate intestinal health, while revealing preliminary insights into foraging ecology, microbiome, and diet.

**Electronic supplementary material:**

The online version of this article (10.1186/s12864-019-5813-z) contains supplementary material, which is available to authorized users.

## Background

Difficulties in obtaining nucleic acids from representative samples make it necessary to implement non-invasive approaches to explore genetic traits in wild animals and humans [1, 2]. In wild apes, non-invasive genotyping methods from feces have been invaluable in determining long term variation in social group dynamics, estimating population abundance, and performing individual tracking [3–7]. Other applications of feces-based, noninvasive genetic methods in wild non-human primates include inferences on evolutionary history [8], seed dispersal patterns [9], and assessment of diet [10–12]; all facilitated by advances in high throughput genomic methods [13].

The application of other noninvasive genetic methods from fecal samples, such as transcriptome analyses, more directly reflective of intestinal immune or metabolic phenotype at the moment of sample collection, are more challenging [14]. Apart from difficulties in obtaining representative tissue samples, significant challenges associated with these analyses include preservation and integrity of samples and nucleic acids in field conditions, contamination with other biological materials in feces (e.g. microbes, dietary materials) and hence, inability to detect nucleic acid signals reflecting animal physiological status. Nonetheless, transcriptomic (mRNA) analyses from fecal samples in humans and animals, targeting intestinal epithelial cells (IECs) via RT-qPCR or RNA-seq, have been previously used to monitor inflammatory biomarkers during diarrhea [15], to profile intestinal metabolism and immunity after birth [16, 17], to assess colon cancer risk [18], and to monitor mucosal transcriptomics in response to non-steroidal anti-inflammatory drug enteropathy [19]. In addition, other techniques, such as droplet digital PCR, have been useful in detecting transcriptional markers of inflammation from human fecal samples [20].

Thus, transcriptomic analyses from fecal samples can be a promising, non-invasive approach to assess intestinal function, when obtaining tissue samples is a challenging task. However, this approach is much less common in wild or captive animals, or human populations living in challenging field conditions, where this noninvasive analysis tool could be invaluable in monitoring for physiological and health status in the context of host health and conservation. Therefore, this study focuses on a preliminary assessment of this noninvasive approach to profile intestinal function in wild primates and human populations in situ. To that end, we validate RNA-seq analyses from fecal samples, with the goal of quantifying differential intestinal gene expression patterns between wild western lowland gorillas (*Gorilla gorilla gorilla*) and a human forager population; the BaAka hunter-gatherers, from the Dzanga Sangha Protected Areas, Central African Republic. We chose these two closely related, but different primate species, expecting to detect wide distinctions in intestinal gene expression, while validating the potential usefulness of the technique for monitoring health and physiological status in habituated or captive wild primates, and isolated human populations when repeated sampling is possible.

The RNA-seq pipeline followed herein sought to preserve the integrity of intestinal epithelial cells in field conditions, while maximizing the amount of transcripts (mRNA reads) obtained by comparing different eukaryotic mRNA enrichment methods (poly(A) mRNA enrichment and/or rRNA depletion). Our data showed that although the proportion of gorilla and human mRNA signals recovered from fecal samples via RNA-seq is significantly small, differential immune and metabolic gene expression patterns denoting intestinal functional distinctions between the two closely related primate species can still be detected.

## Results

### Finding recovery of human and gorilla mRNA reads from fecal samples using poly(a) mRNA enrichment plus rRNA depletion vs rRNA depletion alone

To evaluate two methods for eukaryotic mRNA enrichment from fecal samples, we conducted RNA-seq in 4 sample sets (1 from gorilla and 3 from humans) using poly(A) mRNA enrichment plus rRNA depletion and rRNA depletion alone, generating between 4.5–16.7 M paired end reads (Table 1). After quality control and alignment of pair-end reads to the latest genome drafts for *G. g. gorilla* (gorGor4.1/gorGor4) and *H. sapiens* (GRCh38/hg38), the percentage of reads aligning to the respective host genomes ranged from 0. 44 to 1.41% for samples undergoing poly(A) mRNA enrichment plus rRNA depletion. In contrast, samples in which only rRNA depletion alone was used showed from 0.04 to 0.27% alignment rate to the target genomes. Thus, the combined method showed from 11 to 15 times more mappability to the host genomes (paired t-test, *P* = 0.02), while effectively removing rRNA (0.0 to 4.4%) (Additional file 1: Table S1).

**Table 1:** Evaluation of mRNA enrichment methods using RNA-seq in four sample set. Read statistics along with the % of alignment to *G. g. gorilla* and *H. sapiens* genomes

Samples	Groups	Number of paired-end reads	Remaining reads after quality filtering	Singletons	Low quality reads	Low quality (%)	Total reads aligned using Kallisto	Ratio OLIGO:RIBO	Alignment (%)
KOTO-OLIGO	Haman	13,796,106	13,340,097	0	456,009	3.31	59,616	9.13	0.45
KOTO-RIBO	Human	14,066,624	13,695,055	0	371,569	2.64	6532		0.05
MBUSA-OLIGO	Human	16,559,894	14,492,534	1,269,002	798,358	4.82	103,239	11.85	0.71
MBUSA-RIBO	Human	16,775,019	15,644,483	845,112	285,424	1.70	8714		0.06
IYIKI-OLIGO	Human	4,992,286	4,390,932	437,374	163,980	3.28	57,579	7.41	1.31
IYIKI-RIBO	Human	5,677,816	5,159,806	388,422	129,588	2.28	7772		0.15
MALUI-OLIGO	Gorilla	4,541,457	4,086,161	334,319	120,977	2.66	57,810	5.02	1.41
MALUI-RIBO	Gorilla	4,749,986	4,193,129	418,094	138,763	2.92	11,506		0.27

Signletons: Number of high quailty reads for which the correspoding pairs were discarded during quality filtering

Ratio OLIGO:RIBO: Ratio of aligned reads using poly(A) mRNA enrichment plus rRNA depletion vs rRNA depletion alone

Alignment rate: Number of Kallisto aligned reads**/**number of high quality reads

### Non-host RNA-seq reads in the fecal samples primarily map to the microeukaryotes

**Fig. 1:**
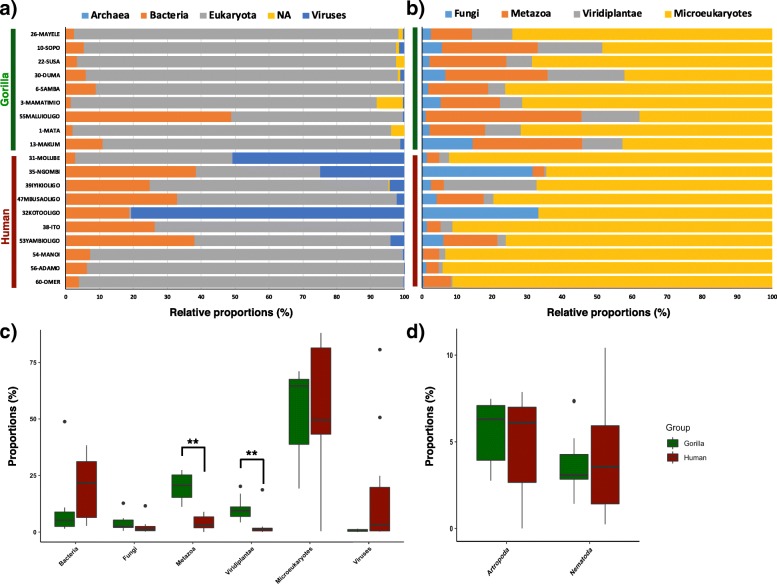
Taxonomic profiling of non-host mRNA reads, using BLAST against the NR database. BLAST on 10,000 randomly selected unmapped reads from each sample: **a**) Percentage relative proportions of major taxonomic groups, **b**) Percentage relative proportions of other eukaryotic hits (excluding gorillas and humans), **c**) Percentage relative proportions of all taxonomic groups identified from gorilla and human samples, **d**) Percentage relative proportion of Arthropods and Nematodes

As poly(A) enrichment plus rRNA depletion yielded the most mappability to human and gorilla genomes, we used this approach for RNA-seq analyses on *n* = 10 fecal samples of BaAka foragers and *n* = 9 samples of wild gorillas, with sequencing depth varying from 2.2 million to 21. 52 million in all samples (Additional file 1: Table S1). However, before proceeding with differential gene expression analyses, we first determined the identity of reads not mapping to the target genomes. To that end, 10,000 randomly selected unmapped reads from each sample were aligned against the NCBI NR database. A strict identity cut-off was used to achieve maximum specificity and hence a large proportion of non-host reads remained unclassified (78.08–98%) (Additional file 2: Table S2). Out of all classified reads, most were of eukaryotic origin (~ 74.77 ± 26.16%) followed by reads mapping to bacteria (~ 15.13 ± 15.06%) (Fig. 1a, Additional file 1: Table S1). Upon closer inspection to the eukaryotic fraction, the majority of reads mapped to microeukaryotes (~ 53.4 ± 23.9% of all eukaryotic reads, excluding gorillas and humans), followed by reads mapping to metazoans (~ 11.6 ± 9.33%), plants (~ 6.4 ± 6.5%) and fungi (~ 3.2 ± 3.5%) (Fig. 1b, Additional file 1: Table S1). Of note, gorillas harbored significantly higher number of reads mapping to metazoans (q = 2.17e-05, Wilcoxon signed-rank test) and plants (q = 0.001, Wilcoxon signed-rank test), and tended to have higher percentage of reads mapping to fungi (q = 0.09, Wilcoxon test). Humans tended to show higher percentage of reads mapping to bacteria compared to gorilla (q = 0.07, Wilcoxon signed-rank test) (Fig. 1c). Further inspection of the metazoan-derived reads indicated similar proportion of reads mapping to arthropods (q = 0.68, Wilcoxon signed-rank test) and nematodes (q = 0. 51, Wilcoxon signed-rank test) in both gorillas and humans (Fig. 1d).

### Differential expression profiles in IECs of gorillas and humans

**Fig. 2:**
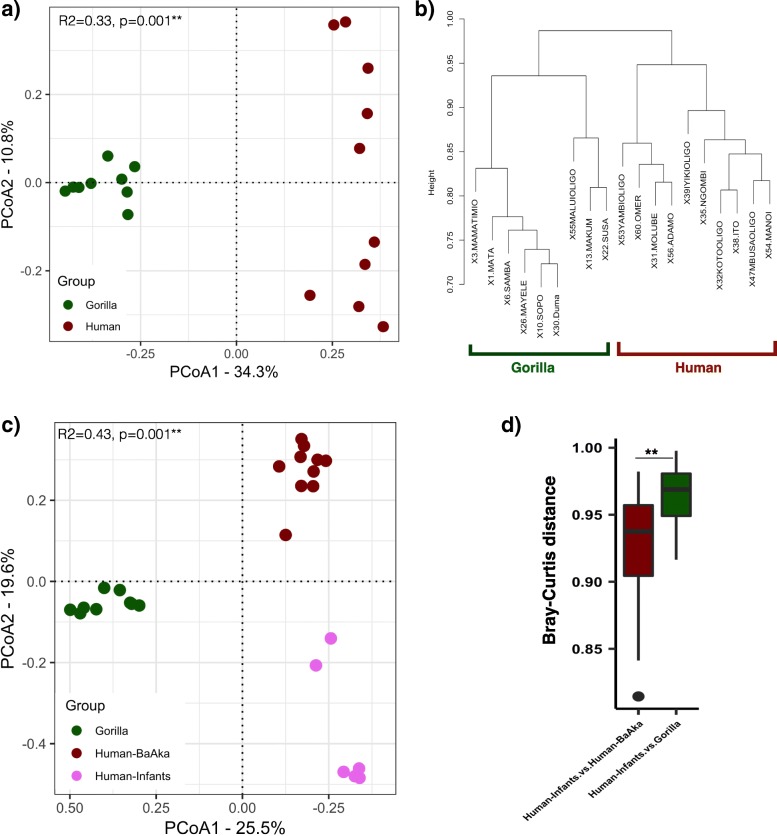
Comparison of human and gorilla transcriptomic data from fecal samples using expression profile of total 15,602 common or orthologous genes: **a**) Principal coordinate analysis based on Bray-Curtis distances, **b**) Hierarchical cluster analysis using hclust function based on the distances calculated using binary distance measure, **c**) Comparison of human-BaAka, gorilla and human-infants (downloaded) transcriptomic data using principal coordinate analysis based on Bray-Curtis distances, **d**) Bray-Curtis distances between these groups

The percentage of reads mapping to human genomes in BaAka fecal samples ranged from 0.40 to 4.99% (1.37 ± 1.36%); while in gorilla fecal samples, the mapping rate to gorilla genomes ranged from 0.26 to 3.1% (1.37 ± 1.00%). Thus, no differences in fecal mappability between gorillas and human were detected (t-test, *p* = 0.99). From the alignments, transcripts per million (TPM) values for 45,717 gorilla transcripts and 179,974 human transcripts were obtained and then a non-redundant (NR) set of 15,602 common genes, based on gene symbol, and known function in both species, was identified. This NR gene set was used for all downstream gene expression comparisons between humans and gorillas using the DESeq2 R package [21, 22]. A principal coordinate analysis based on Bray-Curtis distances showed significant differential gene expression patterns in the gastrointestinal tract between humans and gorillas (PERMANOVA, R^2^ = 0.33, *p* < 0.001) (Fig. 2a), which was further supported by a hierarchical clustering analysis (Fig. 2b). Furthermore, to validate these differential gene expression patterns, we downloaded RNA-seq data targeting IECs shed in fecal samples of human infants. This analysis showed that intestinal gene expression profiles in fecal samples of human infants are closer to those seen in the BaAka hunter-gatherers, based on Bray-Cutis distances (Principal coordinate analysis, PERMANOVA, R^2^ = 0.43, p < 0.001, Wilcoxon rank sum test, p < 0.001, Fig. 2c-d).

**Fig. 3:**
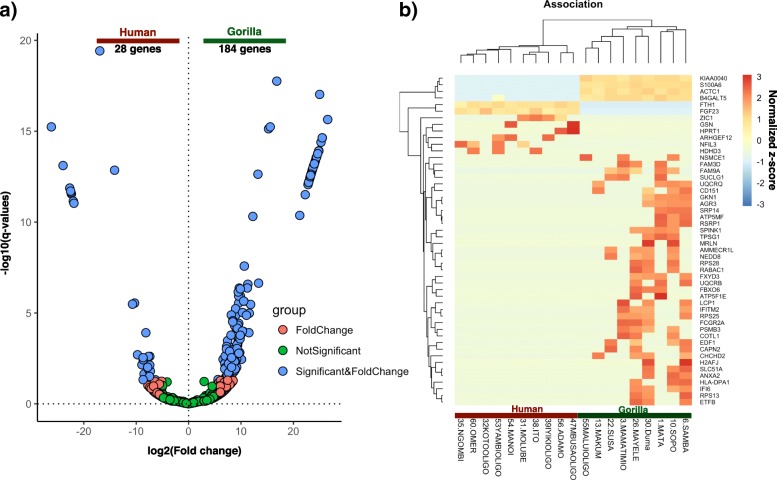
Identification of differentially expressed genes from IECs of gorillas and humans in fecal samples: **a**) Volcano plot of differentially expressed genes. A total 212 genes showed significant differential expression: 184 up-regulated, and 28 down-regulated, **b**) Heatmap representation of top 50 differentially expressed genes based on q-value <= 3.09E-12 and log2 fold change> = 13, between human and gorilla samples. Each row in the heatmap represents gene symbols, whereas each column in the heatmap represents sample names. Color gradient scaling represents the normalized z-scores

**Fig. 4:**
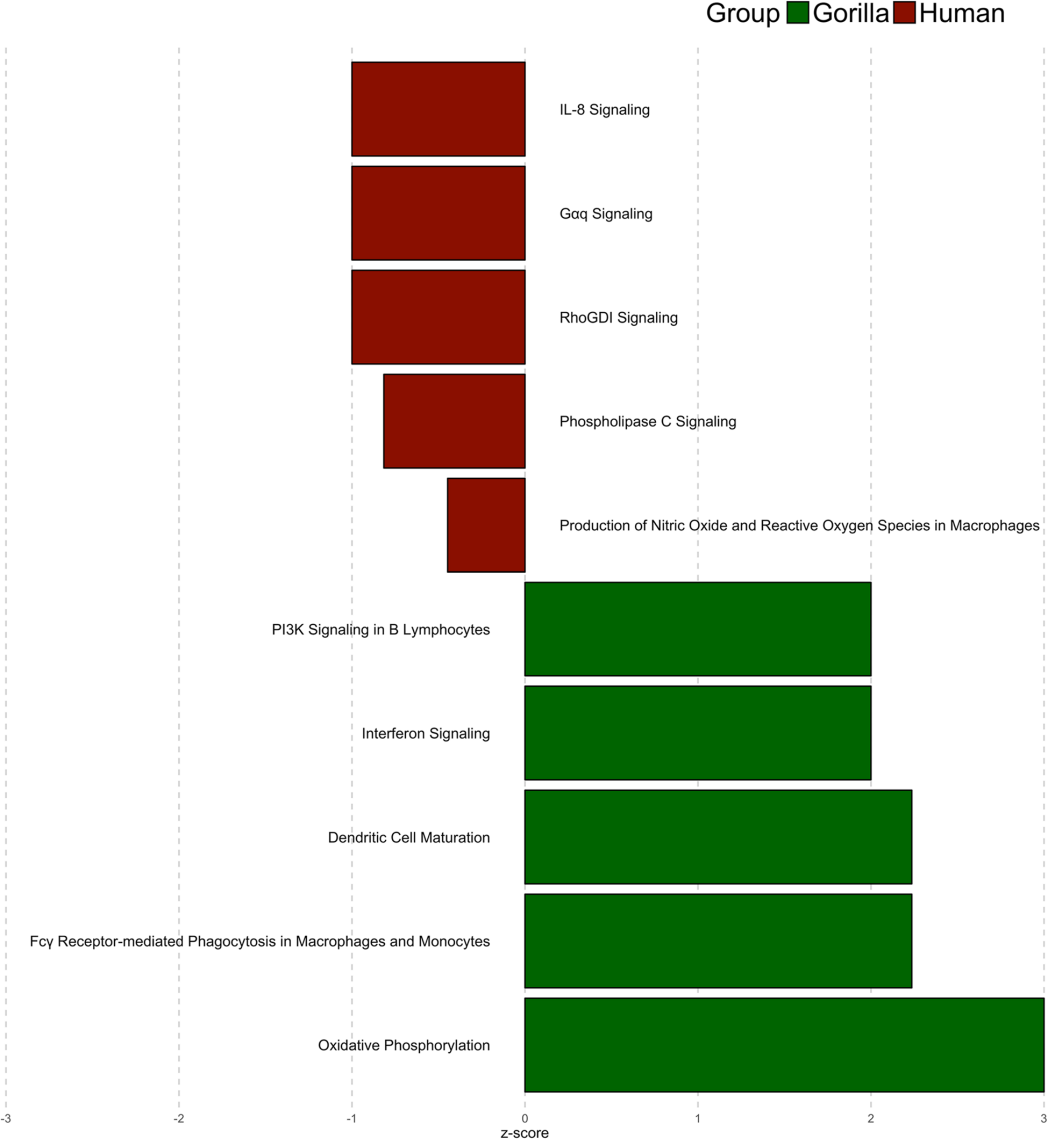
Pathway analysis (Ingenuity IPA software) was performed using differentially expressed genes. Top 5 up-regulated and top-5 down regulated pathways selected based on the IPA provided z-scores

Fold change analyses on the total NR gene set supported by false discovery rate-adjusted q-values (Benjamini & Hochberg correction) identified 212 genes showing significant differential expression between gorillas and the BaAka foragers. One hundred eighty four genes were up-regulated in gorillas while only 28 genes were up-regulated in the BaAka foragers (> 5 fold, q < 0.05, Fig. 3a). The top 50 differentially expressed genes were used to construct a heatmap showing a pool of markers involved in diverse regulatory functions (Fig. 3b and Table 2). Additionally, 26 gene markers specific to gastrointestinal tract metabolism and immune responses were identified (Additional file 3: Table S3). The biological relevance of the differentially expressed genes detected in gorillas and humans was determined using a functional gene set enrichment analysis, as implemented in Ingenuity Pathway Analysis [23]. For this analysis, differentially expressed genes with an FDR q-value cut-off < 0.05 between the two groups were considered. This procedure identified 23 pathways (based on the z-scores) significantly regulated between gorillas and humans (Top 5 upregulated and downregulated pathways in each species can be seen in Fig. 4, all pathways in Table 3). The most upregulated pathway in gorillas was oxidative phosphorylation (*ATP5F1E, ATP5MC3, ATP5MF, ATP5PD, COX5B, COX6B1, SDHB, UQCRB* and *UQCRQ*), followed by phagocytosis in macrophages and monocytes (e.g., *ACTA1, ACTB, ACTC1, ARPC3* and *FCGR2A*), dendritic cell maturation (e.g., *FCGR2A, IL32, LEP, NFKBIA* and *PLCD3*), interferon signaling (e.g., *IFI6, IFITM1*, *IFITM2* and *IFNGR2*) and PI3K signaling in B lymphocytes (e.g., *ATF3, CHP1, NFKBIA* and *PCLD3*) (Fig. 4 and Table 3). In contrast, humans exhibited highly up-regulated expression of genes associated with IL-8 signaling (e.g., *GNB1, LASP1* and *RHOB*), G-protein (*GNB1* and *RHOB*), RhoGDI (e.g., *ARHGEF12, GNB1* and *RHOB*) and phospholipase C (e.g., *ARHGEF12, GNB1* and *RHOB*) signaling and production of nitric oxide and reactive oxygen species in macrophages (e.g., *RHOB*) (Fig. 4 and Table 3).

**Table 2:** List of selected top 50 significantly differentially expressed genes from gorilla and human fecal RNA-seq

Ensembl Id	baseMean	log2FC	lfcSE	pvalue	padj	Gene symbol	entrez ID	Gene name
ENSG00000167996	11,610.8	−16.95	1.69	1.30E-23	3.81E-20	FTH1	2495	ferritin heavy chain 1
ENSG00000159251	10,736.4	16.84	1.76	1.20E-21	1.75E-18	ACTC1	70	actin, alpha, cardiac muscle 1
ENSG00000183304	61.38	25.02	2.68	9.69E-21	9.46E-18	FAM9A	171,482	family with sequence similarity 9 member A
ENSG00000185201	183.02	26.54	2.96	3.09E-19	2.26E-16	IFITM2	10,581	interferon induced transmembrane protein 2
ENSG00000158470	18,496.9	15.59	1.77	1.18E-18	5.77E-16	B4GALT5	9334	beta-1,4-galactosyltransferase 5
ENSG00000152977	704.66	−26.19	2.97	1.10E-18	5.77E-16	ZIC1	7545	Zic family member 1
ENSG00000235750	3610.92	15.27	1.74	1.76E-18	7.37E-16	KIAA0040	9674	KIAA0040
ENSG00000227877	91.61	25.53	2.96	6.52E-18	2.32E-15	MRLN	100,507,027	myoregulin
ENSG00000089356	85.74	25.50	2.96	7.12E-18	2.32E-15	FXYD3	5349	FXYD domain containing ion transport regulator 3
ENSG00000280831	72.47	25.28	2.96	1.38E-17	4.04E-15	RPS25	6230	ribosomal protein S25
ENSG00000169605	64.76	24.87	2.96	4.44E-17	1.18E-14	GKN1	56,287	gastrokine 1
ENSG00000164405	52.34	24.83	2.96	5.02E-17	1.23E-14	UQCRQ	27,089	ubiquinol-cytochrome c reductase complex III subunit VII
ENSG00000126709	45.85	24.65	2.96	8.44E-17	1.72E-14	IFI6	2537	interferon alpha inducible protein 6
ENSG00000233927	45.61	24.64	2.96	8.67E-17	1.72E-14	RPS28	6234	ribosomal protein S28
ENSG00000107223	53.30	24.63	2.96	8.81E-17	1.72E-14	EDF1	8721	endothelial differentiation related factor 1
ENSG00000144233	39.76	24.44	2.96	1.53E-16	2.68E-14	AMMECR1L	83,607	AMMECR1 like
ENSG00000173467	39.14	24.43	2.96	1.56E-16	2.68E-14	AGR3	155,465	anterior gradient 3, protein disulphide isomerase family member
ENSG00000129559	38.07	24.39	2.96	1.77E-16	2.77E-14	NEDD8	4738	neural precursor cell expressed, developmentally down-regulated 8
ENSG00000106153	40.39	24.38	2.96	1.80E-16	2.77E-14	CHCHD2	51,142	coiled-coil-helix-coiled-coil-helix domain containing 2
ENSG00000169189	36.70	24.35	2.96	2.00E-16	2.93E-14	NSMCE1	197,370	NSE1 homolog, SMC5-SMC6 complex component
ENSG00000163541	32.88	24.20	2.96	3.04E-16	4.24E-14	SUCLG1	8802	succinate-CoA ligase alpha subunit
ENSG00000198643	32.08	24.14	2.96	3.59E-16	4.78E-14	FAM3D	131,177	family with sequence similarity 3 member D
ENSG00000275903	29.48	24.03	2.96	4.88E-16	6.22E-14	PSMB3	5691	proteasome subunit beta 3
ENSG00000177697	36.53	23.99	2.96	5.34E-16	6.26E-14	CD151	977	CD151 molecule (Raph blood group)
ENSG00000163959	26.61	23.91	2.96	6.79E-16	7.65E-14	SLC51A	200,931	solute carrier family 51 alpha subunit
ENSG00000196914	49.35	−23.95	2.97	7.09E-16	7.69E-14	ARHGEF12	23,365	Rho guanine nucleotide exchange factor 12
ENSG00000136167	26.11	23.87	2.96	7.58E-16	7.86E-14	LCP1	3936	lymphocyte cytosolic protein 1
ENSG00000241468	25.70	23.86	2.96	7.78E-16	7.86E-14	ATP5MF	9551	ATP synthase membrane subunit f
ENSG00000182718	24.11	23.77	2.96	1.00E-15	9.77E-14	ANXA2	302	annexin A2
ENSG00000164266	38.51	23.73	2.96	1.12E-15	1.06E-13	SPINK1	6690	serine peptidase inhibitor, Kazal type 1
ENSG00000156467	22.80	23.69	2.96	1.24E-15	1.14E-13	UQCRB	7381	ubiquinol-cytochrome c reductase binding protein
ENSG00000140319	21.51	23.60	2.96	1.58E-15	1.40E-13	SRP14	6727	signal recognition particle 14
ENSG00000118972	1630.18	−14.12	1.77	1.62E-15	1.40E-13	FGF23	8074	fibroblast growth factor 23
ENSG00000110700	20.79	23.57	2.96	1.72E-15	1.44E-13	RPS13	6207	ribosomal protein S13
ENSG00000124172	18.93	23.45	2.96	2.43E-15	1.97E-13	ATP5F1E	514	ATP synthase F1 subunit epsilon
ENSG00000105404	18.29	23.39	2.96	2.81E-15	2.22E-13	RABAC1	10,567	Rab acceptor 1
ENSG00000143226	17.72	23.36	2.96	3.10E-15	2.33E-13	FCGR2A	2212	Fc fragment of IgG receptor IIa
ENSG00000197956	886.17	13.24	1.68	3.08E-15	2.33E-13	S100A6	6277	S100 calcium binding protein A6
ENSG00000116663	16.94	23.29	2.96	3.68E-15	2.70E-13	FBXO6	26,270	F-box protein 6
ENSG00000105379	16.11	23.22	2.96	4.51E-15	3.22E-13	ETFB	2109	electron transfer flavoprotein beta subunit
ENSG00000246705	15.06	23.13	2.96	5.68E-15	3.96E-13	H2AFJ	55,766	H2A histone family member J
ENSG00000206291	15.77	22.97	2.96	8.90E-15	5.79E-13	HLA-DPA1	3113	major histocompatibility complex, class II, DP alpha 1
ENSG00000103187	12.75	22.91	2.96	1.04E-14	6.51E-13	COTL1	23,406	coactosin like F-actin binding protein 1
ENSG00000117616	13.04	22.91	2.96	1.04E-14	6.51E-13	RSRP1	57,035	arginine and serine rich protein 1
ENSG00000162909	12.50	22.79	2.96	1.41E-14	8.59E-13	CAPN2	824	calpain 2
ENSG00000148180	19.65	−22.67	2.97	2.24E-14	1.34E-12	GSN	2934	gelsolin
ENSG00000165030	17.54	−22.51	2.97	3.41E-14	1.96E-12	NFIL3	4783	nuclear factor, interleukin 3 regulated
ENSG00000165704	16.69	−22.44	2.97	4.10E-14	2.31E-12	HPRT1	3251	hypoxanthine phosphoribosyltransferase 1
ENSG00000119431	15.72	−22.36	2.97	5.06E-14	2.80E-12	HDHD3	81,932	haloacid dehalogenase like hydrolase domain containing 3
ENSG00000116176	13.05	22.26	2.96	5.70E-14	3.09E-12	TPSG1	25,823	tryptase gamma 1

log2FC: log2FoldChange (Gorilla/human)

lfcSE: logfoldchangeStandard Error

padj: Adjusted *p* value

**Table 3:** Differentially regulated pathways between gorilla and humans identified using Ingenuity Pathway Analysis

Ingenuity Canonical Pathways	-log(*p*-value)	Ratio	z-score	Molecules
Oxidative Phosphorylation	6.02E+ 00	8.26E-02	3	COX6B1,SDHB,ATP5PD,COX5B,ATP5MF,ATP5MC3,UQCRQ,ATP5F1E,UQCRB
Fcγ Receptor-mediated Phagocytosis in Macrophages and Monocytes	2.72E+ 00	5.38E-02	2.236	FCGR2A,ACTB,ARPC3,ACTC1,ACTA1
Dendritic Cell Maturation	1.42E+ 00	2.58E-02	2.236	PLCD3,NFKBIA,LEP,FCGR2A,IL32
Interferon Signaling	3.44E+ 00	1.11E-01	2	IFNGR2,IFI6,IFITM2,IFITM1
PI3K Signaling in B Lymphocytes	1.39E+ 00	2.94E-02	2	PLCD3,ATF3,NFKBIA,CHP1
EIF2 Signaling	9.25E+ 00	7.05E-02	1.89	ATF3,ACTB,RPS23,RPLP2,RPL23,RPL10A,RPS28,EIF1,RPS13,RPS25,RPS15A,RPL36,RPL31,ACTC1,ACTA1,RPLP0
RhoA Signaling	3.74E+ 00	5.65E-02	1.633	ARHGEF12,NRP2,CFL1,ACTB,ARPC3,ACTC1,ACTA1
Regulation of Actin-based Motility by Rho	4.63E+ 00	7.78E-02	1.342	RHOB,CFL1,ACTB,ARPC3,GSN,ACTC1,ACTA1
HMGB1 Signaling	1.98E+ 00	3.60E-02	1.342	HMGB1,CXCL8,KAT6B,RHOB,IFNGR2
Integrin Signaling	3.60E+ 00	4.11E-02	1.134	ARF5,RHOB,ACTB,ARPC3,ZYX,CAPN2,GSN,ACTC1,ACTA1
Signaling by Rho Family GTPases	3.16E+ 00	3.57E-02	1.134	GNB1,CDH1,ARHGEF12,RHOB,CFL1,ACTB,ARPC3,ACTC1,ACTA1
ILK Signaling	2.56E+ 00	3.55E-02	1.134	PPP2CB,CDH1,RHOB,CFL1,ACTB,ACTC1,ACTA1
Actin Cytoskeleton Signaling	4.10E+ 00	4.29E-02	1	ARHGEF12,WASF2,CFL1,ACTB,FGF23,ARPC3,GSN,ACTC1,ACTA1,IQGAP3
Dopamine-DARPP32 Feedback in cAMP Signaling	3.01E+ 00	4.27E-02	1	PLCD3,PPP2CB,KCNJ2,KCNJ14,CHP1,PPP1R11,KCNJ3
Death Receptor Signaling	1.93E+ 00	4.30E-02	1	NFKBIA,ACTB,ACTC1,ACTA1
Tec Kinase Signaling	1.64E+ 00	2.94E-02	1	GNB1,RHOB,ACTB,ACTC1,ACTA1
Sirtuin Signaling Pathway	2.16E+ 00	2.74E-02	0.816	SCNN1A,CXCL8,CDH1,SDHB,SLC2A1,TUBA4B,HMGCS2,ATP5F1E
Neuroinflammation Signaling Pathway	1.12E+ 00	1.93E-02	0.447	HMGB1,CXCL8,CHP1,IFNGR2,GLUL,KCNJ3
Production of Nitric Oxide and Reactive Oxygen Species in Macrophages	1.42E+ 00	2.58E-02	−0.447	PPP2CB,NFKBIA,RHOB,IFNGR2,PPP1R11
Phospholipase C Signaling	1.54E+ 00	2.46E-02	−0.816	GNB1,PLCD3,ARHGEF12,RHOB,FCGR2A,CHP1
RhoGDI Signaling	5.12E+ 00	5.65E-02	−1	GNB1,CDH1,ARHGEF12,WASF2,RHOB,CFL1,ACTB,ARPC3,ACTC1,ACTA1
Gαq Signaling	1.73E+ 00	3.11E-02	−1	GNB1,RGS2,NFKBIA,RHOB,CHP1
IL-8 Signaling	1.35E+ 00	2.46E-02	−1	GNB1,CXCL8,CDH1,RHOB,LASP1

z-score(+): upregulation in gorilla, whereas z-score(−): upregulation in humans

## Discussion

Here, we attempted to maximize functional genomic information from the intestinal tract of wild lowland gorillas and the BaAka foragers from the Dzanga Sangha Protected Areas in the Central African Republic. Our main goal was to determine if the non-invasive RNA-seq approach would detect regulatory signals under selection in the gastrointestinal tract of two closely related primate species, potentially allowing us to answer critical questions on gut physiology of free-ranging gorillas and syntopically living humans. The main obstacle faced by this method was the recovery of sufficient mammalian transcripts from human and gorilla fecal samples. However, as healthy humans can shed approximately 10 billion intestinal cells per day in feces, it has been suggested that detecting mRNA signals from intestinal cells in feces is possible [24, 25].

Despite success in recovering information from IECs and immune cells, percent mappability obtained in this dataset is still lower in comparison to the mappability obtained in human studies (range 0.7–37.9%, mean = 9.21 ± 13.6%) [17]. These differences in mappability may be due to distinctions in sample processing and storage; for instance, we did not perform any bowel fluid preparation to filter out fecal debris nor use a direct poly(A) RNA enrichment extraction kit as described previously [26]. Also, we could not collect fecal samples immediately after voiding, which promotes cellular death. In addition, although we only selected RNA samples of high quality (2 to 5μg of RNA, with a RIN from 7 to 8), RNA purity may have also affected the number of host-derived transcripts obtained. To address these issues, we attempted to test the combined effect of rRNA depletion plus poly(A) enrichment, which improved mammalian genome mappability. In this regard, it has been shown that poly(A) enrichment and rRNA depletion provide similar rRNA removal efficiency, coverage, mappability and transcript quantification, besides providing a less biased coverage of 3′ ends in genes [27]; however, increasing sequencing effort (~ 4 times sequencing depth) may be necessary when using rRNA depletion alone at the same exonic coverage [28]. This is consistent with the increased mappability reached herein, using the combined rRNA depletion and poly(A) enrichment method, as opposed to rRNA depletion alone, at the same sequencing depth. Nonetheless, this combined approach can be considerably more costly compared to using RNA depletion alone. As such, approaches that are gene-centric and that rely on either micro-arrays or RT-qPCR to target gene markers of gastrointestinal disease [29], nutrition [30], and immune and metabolic health [31] may be more cost effective. However, targeted approaches are unable to detect additional information on extrinsic host factors (non-host derived) such as diet and microbiome..

For instance, our results showed that from all the mRNA reads unmapped to gorilla and human genomes, those of plants, metazoa and fungi were more prevalent in gorillas than in the BaAka fecal samples. Thus, non-host RNA-seq reads, as detected here, could potentially offer preliminary insights on dietary behaviors and microbiome. For example, it is a fact that, compared with humans, wild gorillas rely significantly more on plant-based diets [32]. Also, gorillas have been reported to have specialized gut fungal populations to process and ferment highly fibrous and complex foods, an adaptation believed to be of less importance for humans [32]. The observation that both gorillas and the BaAka humans showed similar proportions of arthropod-derived reads is more intriguing; but it may be connected with similar patterns of consumption of insects and insect-derived products by both gorillas and human foragers [33–36]. Likewise, similar prevalence of gastrointestinal nematode infections have been reported by our group in these same gorilla groups and human populations previously [37]. Nonetheless, we were unable to determine why the proportion of all nematode reads was higher in gorillas. This issue is likely a result of the limited taxonomic information that can be recovered from such a small transcript fragment, influence of food processing by the host, and issues related to the technical challenges of this endeavour. Also, due to the liable nature of RNA, DNA-based metabarcoding may be more effective to infer dietary behaviors from feces surveys, in the context of plant and insect consumption [10]. As such, all results related with non-host factors (diet and microbiomes) presented herein warrant further investigation, and comparison with targeted approaches.

Another important consideration of the proposed noninvasive approach is the kind of functional information that can be obtained from exfoliated intestinal epithelial and immune cells in fecal samples. Exfoliated cells in fecal samples may originate from any site along the gastrointestinal tract, including stomach, villus tips in the small intestine and from crypt surfaces at colon level. This information can potentially offer valuable insights on gastrointestinal homeostasis in the context of nutrition and health [25, 38]. However the exact origin of these cells is unknown, and once detached from the extracellular matrix, cells enter a stage of programmed apoptosis and anoikis. Additionally, an important concern is the degradation of RNA coming from small intestinal cells in the lower GI tract and in fecal samples; these limitations may have caused us to miss important expression patterns.

Regardless, the results presented here, along with validation of our data by contrasting fecal mRNA reads of with a previously published human dataset, demonstrate the biological value of fecal RNA-seq to mine for differential gene expression patterns in gut tissues [17, 19]. For instance, we report a higher number of upregulated pathways in gorillas (18 pathways) than in humans (5 pathways), with oxidative phosphorylation in gorillas being the most upregulated pathway in the dataset. Colonic expression of genes involved in energy metabolism, such as oxidative phosphorylation, may be enhanced via the colonic microbiome and its metabolites, specifically butyrate [39]. Thus, it is expected that diets that promote increased fermentation selectively favor this regulatory trait [40]*.* This observation explains the dramatic downregulation of oxidative phosphorylation in the BaAka humans, who incorporate significantly less plant material in their diets. Other gorilla-specific pathways mainly denote maintenance of intestinal barrier integrity, cell architecture and immune functions (Table 3). [A.G2] For example, the upregulation of adaptive immune pathways in gorillas such as phagocytosis in macrophages, dendritic cell maturation and interferon signaling may reflect capacity to recognize and eliminate potential external insults and maintain a balance between tolerance and inflammation in the intestinal environment. Regulatory adaptations to counteract potential pathobionts in the gorilla gut, while tolerating commensals, may be reinforced by reliance on diets that could also inhibit pathogens and stimulate beneficial microbes, such as those rich in phenolics and fermentable dietary substrates (fiber) [41, 42]. These observations may explain why gorillas, compared with the BaAka humans, exhibit down regulation of pathways involved in proinflammation.

For instance, in the BaAka, most upregulated pathways (4 out of the 5 detected) denote increased inflammatory responses. The more upregulated pathway in the BaAka was IL-8 signaling, which mediates recruitment of pro-inflammatory cytokines (CXCL8) to sites of infection [43, 44]. In this regard, it has been reported that African foragers exhibit evolutionary adaptations to counteract increased susceptibility to intestinal infection, by enhancing immune responses to pathogens [37, 45–47]. Other upregulated pathways seen in the BaAka that may support increased responses to pathogens and inflammation are Gɑq signaling and production of nitric oxide (NO) and reactive oxygen (RO) by macrophages. Gɑq signaling plays a role in Paneth cell maturation, intestinal barrier integrity and protection against luminal pathogens by secreting alpha-defensins [48], and NO and RO production in macrophages is correlated with levels of pathogenic infections in monocytes and inflammation [49, 50]. Moreover, phospholipase C signaling is also involved in protection from a hostile luminal environment and tissue restitution after intestinal epithelial cell injury, in conjunction with Gɑq [51, 52]. Based on these results, and the differential expression of pro-inflammatory pathways detected, it is tempting to speculate that humans, in comparison with nonhuman primates are evolutionarily primed for inflammatory responses in response to specific diets, environments or the microbiome; nonetheless, these hypotheses warrants further investigation.

## Conclusions

In summary, we advance a noninvasive approach that evaluates primate intestinal physiology, and that can be used in situ, by measuring gene expression patterns in fecal samples. Although the proportions of IEC mRNA patterns obtained in these conditions are not fully representative, compared to standard RNA-seq approaches, we show that this method has the potential to reveal critical gene expression information when comparing primates exposed to different physiological and environmental conditions. In addition to providing insights into differences among two primate species in intestinal cell function, this approach could reveal fine-grained distinctions among conspecific populations, including differences by age or sex. Broadly, these results have important implications for understanding the immune and metabolic gut environment of humans and animals in filed conditions, when invasive samples are unattainable. In the future, applying this procedure in tandem with methods that provide a more reliable and targeted picture of diet and microbiome (e.g. DNA barcoding, metabolomics and metagenomics) may offer a powerful tool to characterize diet-host-microbe interactions in the context of primate nutrition, health and conservation.

## Methods

### Study site and sampled objects

The study was conducted in Dzanga-Sangha Protected Areas (DSPA) in the Central African Republic. Activities in DSPA are directed by the DSPA administration under the collaborative management of the CAR Government and World Wildlife Fund. The climate is characterized by marked seasonal variation [53], with dry (November–March) and wet (April–October) months [54]. Human population density is low, estimated at one person per square kilometer, with the greatest concentration (60% of people) located in the village of Bayanga [55, 56]. In May 2016, we sampled three habituated gorilla groups (Makumba, Mayeli, Mata) around two permanent Primate Habituation Program (PHP) research camps: Bai Hokou (2° 50 N, 16° 28 E) and Mongambe (2° 55 N, 16° 23 E). The human subjects in the study included personnel working as gorilla trackers (BaAka) hired by the PHP and their female partners. The BaAka males alternated periods of time between the research camps, tracking the gorillas in the Park and their villages. Both trackers and their partners frequently did either day hunts from the villages or longer hunting trips within the DSPA.

### Sample collection and preparation

Fecal samples (1 g) of lowland gorillas and humans were collected within 5 min after defecation and divided in three subsamples (to prevent repeated thawing-freezing if samples need to be analyzed more times) which were immediately in the field preserved in RNAlater (Qiagen, Hilden, Germany) (to prevent repeated thawing-freezing if samples need to be analyzed more times) and left overnight at room temperature. Next day the samples were stored in the mobile freezer powered by solar battery. The necessity to collect the sample immediately after defecation restricts this method only to habituated apes as samples from the unhabituated ones are usually several hours old. In the field, the samples were stored in a mobile freezer powered by solar battery. During the transport to the laboratory in the Czech Republic samples were kept at − 20 °C in a mobile freezer powered by a car battery, while during the international air transport, samples were transported in dry ice. After the arrival to the laboratory in the Czech Republic, samples were stored at − 80 °C and RNA isolated within a month.

### RNA isolation

RNAlater-preserved and frozen feces were thawed on ice to allow collection of approximately 250 mg of feces. The RNA was isolated using the RNeasy Midi Kit (Qiagen, Hilden, Germany) following manufacturer’s instructions. For the cell lysis, β-mercaptoethanol was added to the RLT buffer and RNA was eluted in two steps to two separate tubes using 150 μL of RNAse-free water each time and the isolated RNA was immediately stored at − 80 °C.

### Next-generation sequencing

Total RNA was treated with DNAse and followed by RiboZero rRNA removal kit (bacteria, MRZB12424 Illumina). Samples that yielded from 2 to 5μg of RNA, with a RIN from 7 to 8 were used for downstream applications. RNA was QC’d on the Bioanalyzer using Agilent RNA 6000 Nano Kit (5067–1511), with accepted values of remaining rRNA at 20%. The RNA samples were subjected to mRNA isolation in an effort to capture the host mRNA. The entire sample was used for mRNA isolation using NEBNext poly(A) mRNA Magnetic Isolation Module (NEB E7490L) on the Apollo 324 system. The resulting mRNA was used for generating Illumina libraries using the PrepX RNA-seq for Illumina Library Preparation Kit, 8 sample (Wafergen cat#400039) on the Apollo 324 system with the scriptSeq Index PCR primers (epicenter SSIP1234). Final libraries were side selected at 0.9X. The libraries were QC’s for proper sizing and the absence of adapter dimers on the Bioanalyzer using the Agilent DNA high sensitivity kit (5067–4626) followed by qPCR using the library quantification kit (Kapa Biosystems KK4835) on a 7900HT Fast real-time PCR system (Thermo-Fisher). The libraries were sequenced on the NextSEq 500 at 2*75 base pair read length.

### Gene expression quantification

Quality reports for raw reads were generated using FastQC toolkit. Trimmomatic was used for the trimming of raw reads using “ILLUMINACLIP:$TRIMMOMATIC/adapters/TruSeq2-PE.fa:2:30:10 SLIDINGWINDOW:4:20 MINLEN:36”. Trimmed reads obtained from the gorilla samples were mapped against the gorilla genome and from the human samples were mapped against the Human genome using Kallisto [57]. Aligned counts (TPM values) for each gene from each sample were used for the subsequent analyses. Variance for each was calculated using DESeq2 package after performing the variance stabilizing transformation. Principal coordinate and hierarchical clustering analysis were performed in R. Fold changes and adjusted *p*-values (q-value) for Gorilla vs Human comparison were calculated using DESeq2 at alpha = 0.05 and pAdjustMethod = “BH”. Top 50 differentially expressed genes (q-value < 0.05 & log2 fold change > 10) and having maximum variance were selected for the association analysis using cluster heatmap (distfun = “euclidean”, hclustfun = “complete”).

### Ingenuity pathway analysis

Ingenuity Pathway Analysis (IPA) version 2.0 was used to perform the functional enrichment analysis (QIAGEN Inc., https://www.qiagenbioinformatics.com/products/ingenuity-pathway-analysis/). All differentially expressed genes (q-value < 0.05) were uploaded along with their log2 fold change information. Out of total 212 genes, 184 genes significantly enriched in gorilla (log2 fold change > 5) and 28 genes significantly enriched in humans (log2 fold change > 5) were used for this analysis. The z-score was used to infer the activation state which was calculated using the log2 fold change values of each gene.

### Identification of different taxonomic groups

To identify the taxonomic fractions other than the host genome, 10,000 randomly selected unmapped reads from each sample were aligned against the NCBI NR database using BLAST (default parameters)[58]. The reason for selecting this database was to predict the maximum signals from the non-host RNA reads as a preliminary assessment. Resultant blast best hits were selected using the identity threshold of > 99%. The taxonomic IDs were imported from NCBITaxa using ETE toolkit to obtain the complete taxonomic lineage of each hit [59].

### Dataset downloaded for the comparison

Infants RNA-seq reads for six individual samples were downloaded from the published study [SRA:PRJNA182262] [17]. Total 300 million, 50 bp single end reads were processed using the same pipeline used in our study to generate the expression profiles.

## Additional files

Additional file 1: **Table S1.** Read statistics, percentage alignments and percentage proportions of non-host RNA-seq reads (XLSX 12 kb)

Additional file 2: **Table S2.** Percentage proportions of classified and unclassified reads out of 10,000 randomly selected non-host RNA-seq reads (XLSX 10 kb)

Additional file 3: **Table S3.** Comparative expression of specific markers to gastrointestinal tract and immune responses from gorilla and human fecal RNA-seq (XLSX 12 kb)

## Data Availability

The datasets generated for the current study have been deposited in the NCBI BioProject database under project number PRJNA541574 and the infant dataset used for the comparative analysis was downloaded from accession number PRJNA182262.

## Abbreviations

FDR: False discovery rate
IECs: Intestinal Epithelial Cells
mRNA: Messenger RNA
PI3K: Phosphoinositide 3-kinase
RNA-seq: RNA sequencing
rRNA: Ribosomal RNA
TPM: Transcripts per million
